# Absolute and adjusted CO_2_ equivalent emissions data of EU regions’ energy and heavy industry sectors

**DOI:** 10.1016/j.dib.2025.112320

**Published:** 2025-11-25

**Authors:** Chiara Vagnini, Leticia Canal Vieira, Mariolina Longo, Matteo Mura

**Affiliations:** Department of Management – University of Bologna, Via Terracini 28, 40131 Bologna, Italy

**Keywords:** Industrial decarbonization, Total CO_2_ equivalent emissions, Per-capita CO_2_ equivalent emissions, Industrial GVA-adjusted CO_2_ equivalent emissions, EU Emissions Trading System, NUTS 2, Longitudinal data

## Abstract

This article presents a balanced panel dataset of absolute and adjusted carbon dioxide equivalent (CO_2_-eq) emissions (i.e., total, per capita, and per unit of industrial gross value added) from energy and heavy industry sectors in 238 regions across 27 EU countries over a 13-year period (2008–2020). Secondary data on verified CO_2_-eq emissions aggregated at the Nomenclature of Territorial Units for Statistics (NUTS) 2 level, as defined by EUROSTAT, for the period 2008–2016 were obtained from a previous dataset [1]. Additional CO_2_-eq emissions data for 2017–2020 were extracted at the installation level from the EU Emissions Trading System (ETS) registry. Data extraction was automated through a dedicated script, followed by consistency checks to ensure reliability; the resulting data were then aggregated to the regional level. Demographic data on population (as of 1 January) and economic data on industrial gross value added (GVA, expressed in million euros) were obtained from EUROSTAT at the NUTS 2 level for the entire reference period and incorporated into the dataset. Changes in the NUTS 2 classification during the considered period required dataset harmonization and imputation techniques to address missing values. The dataset has been used in prior research to analyze spatial and temporal patterns of CO_2_-eq emissions [2] and their socio-economic drivers across EU regions [4]. Its transparent and replicable structure ensures reproducibility, facilitates the integration of new emissions and socio-economic data, and supports future studies on regional decarbonization dynamics, emission clustering, and spatial spillover effects.

Specifications Table

**Table:** 

Subject	Earth & Environmental Sciences
Specific subject area	Regional progress towards net-zero targets of EU energy and heavy industry sectors.
Type of data	Table.
Data collection	Secondary data on verified CO_2_-eq emissions aggregated at the NUTS 2 level for the period 2008–2016 were obtained from [1], while additional emissions data for 2017–2020 were collected at the installation level from the EU ETS registry and subsequently aggregated to the NUTS 2 level. Following the methodology of [1], a script was developed to automate data extraction, and consistency checks were performed to ensure reliability. Demographic data on population (as of 1 January) and economic data on industrial gross value added (GVA, expressed in million euros) were obtained from EUROSTAT at the NUTS 2 level for the entire reference period and incorporated into the dataset.
Data source location	For CO_2_ equivalent emissions data: https://union-registry-data.ec.europa.eu/report/welcome For population: https://ec.europa.eu/eurostat/databrowser/view/demo_r_d2jan/default/table?lang=en&category=reg.reg_dem.reg_dempoar For industrial GVA data: https://ec.europa.eu/eurostat/databrowser/view/nama_10r_3gva/default/table?lang=en&category=reg.reg_eco10.reg_eco10brch
Data accessibility	Repository name: Mendeley Data Data identification number: 10.17632/gcmxns5nmk.2 Direct URL to data: https://doi.org/10.17632/gcmxns5nmk.2
Related research article	Vagnini, C., Vieira, L. C., Longo, M., & Mura, M. (2025). Chasing net zero: An exploratory space-time analysis of European regions’ industrial carbon emissions. *Journal of Environmental Management, 391*, 126,466. https://doi.org/10.1016/j.jenvman.2025.126466

## 1 Value of the Data

• The dataset provides a consistent collection of verified CO_2_ equivalent emissions data from industrial facilities across 238 NUTS 2 EU regions, covering the period 2008–2020. By aggregating plant-level data from the EU-ETS registry based on the NUTS established by EUROSTAT, it enables analyses beyond the national scale, capturing the heterogeneity and regional characteristics inherent in EU industrial emissions.
• Researchers can use the dataset to conduct further analyses, replicate existing studies, and enrich their work through additional investigations. Its standardized structure, transparent and replicable methodology, and compatibility with publicly available sources such as EUROSTAT and EU ETS also make it suitable for comparative research and future data extensions. In this regard, it can be complemented with CO_2_-eq emissions data for subsequent years (after 2020) and with additional socio-economic variables, such as institutional factors (e.g., quality of regulations and standards), industrial-specific factors (e.g., energy mix use, the number of facilities, employment levels, technological innovation, and environmental practices), imports and potential exports beyond the EU, and more.
• The dataset lends itself to a variety of methodological approaches that can be further applied to uncover additional insights. Previous studies have already employed it to investigate different aspects of regional decarbonization dynamics across the EU. In particular [2], used the complete dataset to perform an Exploratory Space–Time Data Analysis (ESTDA), assessing spatiotemporal interdependencies in industrial CO₂ emissions regions. This approach allowed to identify spatial and temporal patterns of decarbonization, detect persistent emission clusters, and highlight the existence of polarized regional trajectories. Another study [4] applied spatial econometric models to a subset of the dataset (i.e., total CO_2_-eq emissions and industrial GVA), integrating it with additional socio-economic variables to examine the regional determinants and interregional relationships influencing industrial CO_2_-eq emissions. This methodological framework revealed significant spatial spillovers and temporal persistence in emissions.
• The dataset offers a foundation for future studies. For example, it can support a deeper investigation into the spatial distribution of decarbonization progress across EU regions, the persistence of regional emission patterns, or the policy responsiveness across regions with different levels of industrial development or carbon dependency. In addition, researchers can use it to explore clusters of high or low emitters and evaluate how socioeconomic factors influence industrial carbon performance. The dataset is particularly well suited for research focused on spatial spillovers, transition dynamics, and regional lock-ins.
• The dataset also offers practical value for policymakers, due to its granular, region-level detail and the inclusion of per-capita and GVA-adjusted emissions indicators, which support the design of differentiated climate policies tailored to local contexts. It allows decision-makers to identify regions where emissions remain consistently high and prioritize them for targeted interventions, while also highlighting frontrunner regions that may serve as models or innovation leaders. In addition, the dataset facilitates the monitoring of long-term progress and helps detect potential plateaus in emissions reduction efforts. Its structure enables the identification of regional clusters with similar decarbonization trajectories, even across national borders, offering insights for coordinated policy planning.

## 2 Background

The dataset was originally compiled to support an exploratory spatiotemporal analysis of industrial CO_2_-eq emissions across EU regions. Drawing on existing literature on measuring progress towards EU net-zero targets, as presented in [2], the aim was to capture subnational heterogeneity in CO_2_-eq emissions from hard-to-abate industrial sectors and advance understanding of spatial patterns, disparities, and interdependencies in the areas where these emissions originate.

Using regions as the scale of analysis, the data on CO_2_-eq emissions in million tons, per-capita CO_2_-eq emissions, and CO_2_-eq emissions per unit of industrial GVA allow for a more granular representation of emissions patterns. This reflects the territorial diversity of EU regions not only in terms of environmental impact, but also in demographic characteristics, socioeconomic structures, and levels of industrial development [3,4].

In addition to the published articles by [1,2], and [4], this dataset provides a standardized and reusable source of regional emissions data. It enables further investigation into spatial emission trends, supports methodological development, and facilitates comparative or extended analyses beyond the original research scope.

## 3 Data Description

The files associated with this Data-in-Brief article are provided in Excel format and include:

• A balanced panel dataset of CO_2_-eq emissions (million tons), per-capita CO_2_-eq emissions (ton/person), and CO_2_-eq emissions per unit of industrial GVA (ton/ten thousand euros) for 238 regions over a 13-year period (2008–2020), each reported in a dedicated sheet.
• Complementary demographic and economic variables, namely population on 1 January (number) and GVA at basic prices (million euros) for the industry sector.

Both files report values at the NUTS 2 geographical level. Each NUTS 2 region is listed together with information on its higher territorial levels, namely NUTS 1 and NUTS 0. For each record, both the NUTS code and the corresponding regional label are provided. Finally, the dataset includes yearly values for all three variables across the full observation period, organized in dedicated columns.

## 4 Experimental Design, Materials and Methods

With the aim of analyzing spatiotemporal patterns and regional disparities in CO_2_-eq emissions across the EU, and of assessing progress towards net-zero targets in energy and heavy -industry sectors, we developed a comprehensive balanced panel dataset of CO_2_-eq emissions at the regional level. The dataset covers total emissions (million tons), per-capita emissions (ton/person), and emissions per unit of industrial GVA (ton/ten thousand euros) for 238 NUTS 2 regions across 27 EU Member States over the period 2008–2020.

Secondary data on verified CO_2_-eq emissions aggregated at the NUTS 2 level for the period 2008–2016 were obtained from [1], while additional emissions data for 2017–2020 were obtained from the EU ETS, which publishes annual plant-level greenhouse-gas (GHG) emissions. The EU ETS registry covers >11,000 energy-intensive installations (e.g., oil refineries, steel works, and production of iron, aluminium, metals, cement, lime, glass, ceramics, pulp, paper, cardboard, acids and bulk organic chemicals), accounting for approximately 45 % of the EU’s GHG emissions. Plant-level emissions were expressed as CO_2_ equivalent, translating different gases into a common unit based on global warming potential. In line with the methodology of [1], a script was implemented to automate the extraction of data, and the dataset was subjected to consistency checks to verify its reliability. The extracted data were then aggregated to the NUTS 2 geographical level as defined by EUROSTAT.

The NUTS classification comprises four hierarchical levels: NUTS 0 (Member States), NUTS 1 (major socio-economic regions), NUTS 2 (basic regions for the application of regional policies) and NUTS 3 (small regions such as municipalities or counties). We used the 2016 NUTS classification, following the approach of [1].

Data on population (as of 1 January, unit: number) and industrial GVA at basic prices (unit: million euros) were retrieved from EUROSTAT at the NUTS 2 level. The industry sector is defined here as the aggregation of NACE Rev. 2 sectors B–E (B: Mining and quarrying; C: Manufacturing; D: Electricity, gas, steam and air-conditioning supply; E: Water supply; sewerage; waste management and remediation activities).

Missing values were imputed using the Multivariate Imputation by Chained Equations (MICE) framework, with Classification and Regression Trees (CART) as the imputation algorithm.

The final balanced panel comprises 238 NUTS 2 regions observed annually from 2008 to 2020, yielding 3094 region-year units and, given the three primary variables reported, a total of 9282 observations across variables.

## Limitations

We encountered three main limitations in compiling the dataset. First, we excluded all United Kingdom (UK) regions from the sample. When we collected the data in 2023, UK regions were no longer included in the Eurostat database or in the NUTS classification following the country’s withdrawal from the EU.

Second, the NUTS classification underwent several revisions during our period of interest, involving the creation, split, merger, or boundary change of regions. Following the approach of [1], we adopted the 2016 NUTS classification and harmonized data from earlier and later versions accordingly. In cases where socioeconomic values were missing, we examined whether the region had been subject to reclassification, which allowed us to recover most of the gaps.

Third, data included in the dataset extend only to 2020, as this was the most recent year with verified CO_2_-eq emissions from the EU ETS at the time of compilation.

## Ethics Statement

The authors have read and followed the ethical requirements for publication in Data in Brief and confirm that the current work does not involve human subjects, animal experiments, or any data collected from social media platforms.

## CRediT Author Statement

**Chiara Vagnini:** Writing – original draft, Methodology, Data curation, Conceptualization. **Leticia Canal Vieira:** Writing – review & editing, Conceptualization. **Mariolina Longo:** Writing – review & editing, Supervision, Project administration, Conceptualization. **Matteo Mura:** Writing – review & editing, Supervision, Project administration, Conceptualization.

## Data Availability

Mendeley DataAbsolute and adjusted CO2 equivalent emissions data of EU regions' energy and heavy industry sectors (Original data).
